# The Regulation of the CNS Innate Immune Response Is Vital for the Restoration of Tissue Homeostasis (Repair) after Acute Brain Injury: A Brief Review

**DOI:** 10.4061/2010/151097

**Published:** 2010-08-09

**Authors:** M. R. Griffiths, P. Gasque, J. W. Neal

**Affiliations:** ^1^Deptartment of Medical Biochemistry, University Hospital of Wales, Cardiff University Medical School, Cardiff CF14 4XN, UK; ^2^University Labo. Biochimie et Genetique Moleculaire, Facilities de Science et Technologies, Universite de La Reunion, 15 Avenue Rene Cassin Saint Denis, Ile de la Reunion, BP 7151, 97715, France; ^3^Deptartment of Histopathology, University Hospital of Wales, Cardiff University Medical School, Cardiff CF14 4XN, UK

## Abstract

Neurons and glia respond to acute injury by participating in the CNS innate immune response. This involves the recognition and clearance of “not self ” pathogens and “altered self ” apoptotic cells. Phagocytic receptors (CD14, CD36, TLR–4) clear “not self” pathogens; neurons and glia express “death signals” to initiate apoptosis in T cells.The complement opsonins C1q, C3, and iC3b facilitate the clearance of apoptotic cells by interacting with CR3 and CR4 receptors. Apoptotic cells are also cleared by the scavenger receptors CD14, Prs-R, TREM expressed by glia. Serpins also expressed by glia counter the neurotoxic effects of thrombin and other systemic proteins that gain entry to the CNS following injury. Complement pathway and T cell activation are both regulated by complement regulatory proteins expressed by glia and neurons. CD200 and CD47 are NIRegs expressed by neurons as “don't eat me” signals and they inhibit microglial activity preventing host cell attack. Neural stem cells regulate T cell activation, increase the Treg population, and suppress proinflammatory cytokine expression. Stem cells also interact with the chemoattractants C3a, C5a, SDF-1, and thrombin to promote stem cell migration into damaged tissue to support tissue homeostasis.

## 1. Introduction

Acute ischemic brain infarction and traumatic brain injury share several pathological features, including the disruption of the Blood Brain Barrier (BBB) with entry of systemic inflammatory cells and circulating blood proteins into the brain parenchyma. The reduced blood flow frequently results in hypoxia contributing to neuronal ischemia, inflammation, and apoptosis [1, 2]. The surviving resident brain cells (neurons and glia) are not “professional” immune cells, but contribute to the defence of the brain through the expression of the innate immune response, promoting the clearance of neurotoxic proteins and apoptotic cells from the Central Nervous System (CNS). This stimulates both tissue repair (resolution) and the rapid restoration of tissue homeostasis [3–6].

This review will examine how the CNS innate immune response maintains a critical balance between the protective and potentially harmful effects of activating the innate immune system following acute brain injury, the so-called “double-edged sword” effect [7]. The balance between the destructive and protective effects of the innate immune response must be precisely regulated in order to promote conditions that support brain repair and encourage a return of tissue homeostasis [5, 8, 9].

**Figure 1:**
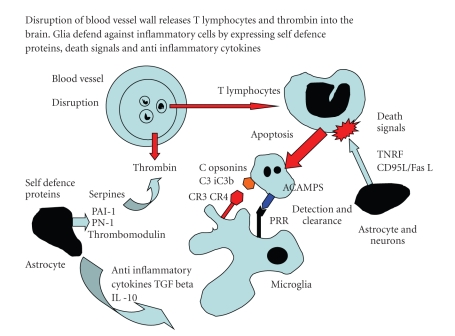
Shows the consequences of disruption of the blood brain barrier BBB. Thrombin is an example of a protein with pathogen protein associated molecular pattern (PPAMPS) released into the neuropil and its neurotoxic effects are countered by the expression of glial “selfdefence” proteins including the serpins (serine protease inhibitors) protease derived glial-nexins PA-1, PN-1. Systemic T cells are identified and targeted by “Death signals” TNF and CD95L/CD95F as expressed by astrocytes and neurons this initiates apoptosis. Apoptotic cells defined as “altered self” by cell surface apoptotic cell associated molecular patterns (ACAMPs) are identified by microglia expressing pattern recognition receptors (PRR) and subsequently cleared from the brain, reducing the severity of the inflammatory response.

The CNS innate immune response relies upon the resident cells (neurons and glia) expressing both phagocytic and scavenger receptors capable of distinguishing “self” (host) from “nonself” (neurotoxic proteins, pathogens, apoptotic cells) and so reduce bystander injury [10–14]. Neurons and glia also express “death signals” to initiate apoptosis in damaged neurons and inflammatory cells, transforming them into “safe targets” for rapid clearance from the CNS by glial cells expressing phagocytic receptors [10, 11]. If apoptotic cells remain undetected and not cleared from inflamed tissues, they will undergo lysis with the release of neurotoxic enzymes, contributing to secondary host tissue necrosis [1]. The components of the complement pathways (CP) include opsonins and chemoattractant proteins that are synthesized by neurons and glia. These two groups of complement proteins facilitate pathogen and apoptotic cell phagocytosis, as well as inflammatory cell migration into areas of tissue damage [10, 11]. The regulation of the destructive arm of the “double-edged sword” is vital and relies upon serpins (selfdefence proteins), regulators of complement activation (RCAs) (sometimes referred to the complement regulatory proteins (CRP) and various neuroimmunoregulatory molecules (NIRegs) such as CD200 and CD47. All these regulators are expressed by glia and neurons [10, 11] (Figure 1). Finally, there is increasing evidence that host stem cells contribute to the immune regulation of tissue inflammation through their interaction with the same brain cells responsible for the CNS innate immune system response [4, 15–17]. 

## 2. The Diverse Talents of the CNS Innate Immune System: Detection and Clearance of “Nonself” Cells and Proteins from the Brain

Neurons and glia are not “professional phagocytes”, but express highly conserved pattern recognition (PRR) and scavenger receptors (SR) [13, 14, 18–26]. These receptors distinguish host “self” from apoptotic cells and pathogens (“nonself”) [14, 18–26] helping to prevent indiscriminate cell death and uncontrolled tissue damage [24, 25]. For example, apoptotic cells express apoptotic cell-associated molecular patterns ACAMPS, such as phosphatidylserine and carbohydrate molecules on their cell surface, whereas these molecules are absent from host cells [27]. ACAMPs represent a group of unique cell surface molecules representing “nonself” and allow apoptotic cells to be distinguished from “self” or host tissues that do not express ACAMPs [8, 9, 23, 28]. Pathogens express pathogen-associated molecular patterns (PAMPs) composed of a lipopolysaccharide (LPS) and other peptidoglycans only found in bacteria cell walls [25, 26]. The neurotoxic proteins (*α* synuclein, mutant prion protein, thrombin, HMGB1, S100, and A4 *β* amyloid) are released from damaged cells and identified as “nonself” because they contain pathogen protein associated molecular patterns PPAMPS [10, 11].

A wide range of PRR and SR are expressed by microglia and astrocytes and contribute to a range of phagocytic pathways poised to remove apoptotic cells, pathogens, and neurotoxic proteins from the CNS, contributing to restoration of tissue homeostasis [10, 11, 14, 22, 29, 30]. The clearance through phagocytosis results in the so-called “nonphlogistic” response and is associated with a subsequent reduction of tissue inflammation and promotion of repair (tissue homeostasis) [8, 10, 11, 30–32]. 

## 3. The Complement System Is Vital for Apoptotic Cell Clearance and Regulation of CNS Inflammation

**Figure 2:**
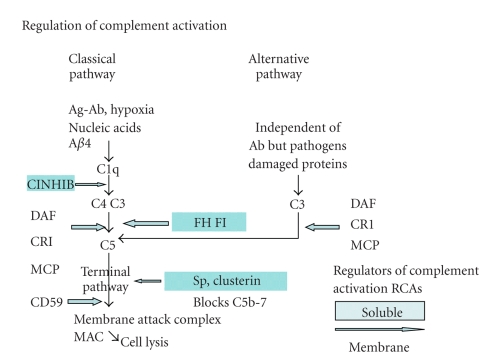
This figure shows a summary of the individual components of the classical and alternative complement pathways (CP), both converge at the C3/C5 step and share a common terminal pathway with eventual generation of the cytolytic protein, membrane attack complex MAC. The membrane bound complement regulatory proteins (CRP) CRI (CD35), DAF decay accelerating factor (CD55), MCP (CD46), CD59 at the sites along the pathway where they inhibit C synthesis. The soluble CRP, C inhib, clusterin, Factor H (FH), Factor I (FI) are also shown.

The CNS innate immune response also involves two of the three C pathways; the classical and alternative C pathways (Figure 2). These two pathways provide the cytolytic membrane attack complex (MAC) and molecules called opsonins that target pathogens and neurotoxic proteins, both identified as “nonself”. The opsonin molecules generated by the CP are able to identify apoptotic cells (altered self), because they express ACAMPS on their cell surface [25, 32, 33]. Glia and neurons express a full range of RCAs (sometimes described as complement regulatory proteins, CRP). These regulators are capable of preventing excessive complement activation and they inhibit MAC-related cytolysis of innocent “host” bystander cells. (For detailed discussion see [32, 34]).

A further immunoregulatory strategy employed by the innate immune system is the expression of “don't eat me” inhibitory signals by glia and neurons. These molecular signals are the so-called Neuroimmunoregulatory proteins (NIRegs) and include CD200 and CD47 [6, 10, 11, 35–37]. These two cell surface molecules and their receptors, CD200R and CD172a, respectively, modulate the activation of inflammatory cells (lymphocytes and activated microglia), to reduce the level of tissue inflammation and contribute to brain tissue repair [38–40] (see Figure 1).

## 4. The Blood Brain Barrier and CSF/Brain Barriers; Vital Barriers to Prevent Systemic Cell Entry and Maintain Immunoprivilege

The mammalian brain is isolated from the systemic circulation by a protective blood brain barrier (BBB) composed of endothelial cells linked by tight junctions and surrounded by the end feet of astrocytes [41]. A further layer of ependymal cells lines the ventricle wall preventing entry of pathogens and inflammatory cells from the CSF into the brain [42, 43].

Within the peri vascular layer and choroid plexus (CPLx) are CD163+ and MHC II+ cells with evidence of PRR expression in the form of CD14 and Toll-like receptors (TLR). These cells and their receptors are capable of detecting pathogens and apoptotic cells in the CSF [21, 44, 45]. Preservation of these physical barriers under physiological conditions contributes to the immuno privileged status of the CNS [41, 46].

## 5. Neurones and Glia Protect the CNS by Regulating the Entry of Inflammatory Systemic Cells into the Brain at the Blood Brain Barrier

The inhibition of cell adhesion to the endothelium prevents the entry of myeloid (neutrophils and lymphocytes) derived cells across the BBB preserving the immuno privileged status of the brain. Neurons are an important source of TGF-*β* [47] and this anti-inflammatory cytokine down regulates both astrocyte and endothelial expression of the C pathways, Monocyte chemoattractant protein-1 (MCP-1), and Vascular cell adhesion molecule VACAM-1, preventing lymphocyte entry into the brain under physiological conditions [48, 49]. Astrocytes are a vital component of the BBB and induce the expression of leukocyte adhesion molecules by endothelial cells [41]. VCAM-1 is a member of the Ig super gene family and a regulator of T lymphocyte transport across the BBB [50]. Brain injury activates microglia with the increased expression of the proinflammatory cytokines TNF-*α* IL-1 *β* and IFN-*γ* which in turn stimulate the expression of VCAM-1, and MCP-1, both capable of increasing T cell entry into the CNS [49, 51]. The NIreg molecule, CD47, is expressed by cerebral endothelium and it regulates the trans-migration of monocytes across the BBB under inflammatory conditions [46]. Further protection at the BBB is provided by the ependymal cell expression of RCA preventing the excessive activation of complement by neurotoxic proteins and apoptotic cells in the CSF and on the ventricular surface of the brain [42].

## 6. Acute Disruption of the Blood Brain Barrier Exposes the Brain to Neurotoxic Systemic Proteins

Acute brain damage includes spontaneous haemorrhage, ischaemic brain infarction, and raised intracranial pressure due to cerebral oedema. A feature common to these events is the disruption of the BBB, permitting entry into the brain of systemic proteins [10]. One systemic protein that enters the brain following haemorrhage is thrombin, a serine protease, vital for blood coagulation. Thrombin is generated in the systemic circulation by cleavage of prothrombin (PT) by factor Xa, the fibrinolytic protein plasminogen is converted to plasmin by the action of tPA (tissue plasminogen activator) [52].

Under physiological conditions, thrombin is prevented from entering the CNS by the intact BBB; however, it is also synthesised in low concentrations by neurons and astrocytes [52, 53]. At low concentrations [50 pM–100 pM], thrombin is important for brain repair as it regulates nerve growth factor synthesis, synaptic outgrowth in adults and tissue remodelling [54]. It also has neuroprotective effects due to its modulation of intracellular calcium and is also protective against both oxygen and glucose deprivation [55–57].

The high concentration (500 nM) of thrombin in the brain following intracerebral haemorrhage ICH and BBB disruption is neurotoxic. The presence of a high concentration of brain thrombin activates both NMDA excitotoxic receptors precipitating seizures and stimulates the protease-activated receptor-1 (PAR-1) that inhibits neurite extension by stimulating astrocyte proliferation. The result of these effects is to prevent neuronal repair [58]. Thrombin is also neurotoxic through its activation of microglia via the JAK2-STAT3 signalling pathway promoting TNF-*α* and NO expression [59]. The brain responds to the neurotoxic levels of systemic proteins such as thrombin by neurons and glia expressing a range of “self-defence proteins”. Amongst them are the serpins (serine protease inhibitors) that are vital for the restoration of tissue homeostasis [5, 10].

## 7. Serpins Are a Family of “Selfdefence” Proteins Expressed by Resident Brain Cells to Defend against Neurotoxic Proteins

The serpins include the antithrombin colligin (Hsp47) located in microglia and astrocytes; the plasminogen activator inhibitor (PAI-1) and protease glial derived nexin-1 (PN-1) both expressed by astrocytes and neurons [52, 53, 60–62]. A nonserpin thrombomodulin (CD141) is expressed by microglia and-endothelium after injury. This molecule reduces thrombin induced neuronal death, underlining the potential of CD141 as a therapeutic agent [63, 64]. 

The serpin, Pigment epithelium derived factor (PEDEF), is selectively trophic for motor neurons, protecting them *in vitro* against glutamate toxicity and also blocking microglial proliferation [65, 66]. PAI-1 and PN-1 are serpins expressed by neurons and astrocytes; both inhibit neurotoxic thrombin formation [62]. Ischaemic brain injury increases TGF-*β* expression and its neuroprotective properties are mediated by a serine protease released from astrocytes. *In vitro* TGF-*α* and TGF-*β* stimulate astrocyte expression of PAI-1 which is responsible for their neuroprotective effects observed following excitotoxic acid injection into the CNS and cerebral ischaemia [6, 67, 68]. The level of glial PN-1 also rises following hippocampal ischaemia and this provides a degree of neuroprotection [69]. In a rat model of stroke, the expression of neuroserpin, an inhibitor of plasminogen, (tPA) is restricted to neurons and astrocytes localised around the penumbra [52, 58, 67, 70]. The experimental administration of neuroserpin reduced infarct volume by inhibiting thrombin synthesis and promoting a neuron survival [70, 71]; see Figure 1. 

## 8. Neurons and Glia Provide “Self-Defence” against the Detrimental Effects of Brain Inflammation

The regulation of microglial activation and inflammatory cytokine synthesis following brain injury is vital, in order to prevent further tissue damage [4, 8, 10, 11]. Neurons and glia are in close communication through a number of signalling pathways and are capable of regulating proinflammatory cytokine expression following brain injury and inflammation [72].

A detailed review of cytokine regulation and tissue repair is not attempted here, but briefly microglia and astrocytes respond to pathological stimuli by adopting a characteristic activated phenotype. This is associated with the expression of a wide range of proinflammatory cytokines including complement (C), tumour necrosis factor (TNF-*α*), the interleukins (IL-5, IL-6, IL-12, IL-1*α*, IL-1*β*), NO (nitrous oxide), and free oxygen radicals (For review see [6]).

One source of the anti-inflammatory regulatory cytokines, IL-10 and TGF-*β* is local astrocytes and neurons [48, 70, 73, 74]. In vitro, IL-10 inhibits LPS stimulated microglial synthesis of IL-2, IL-6, and TNF-*α* by inhibiting expression of the NF kappa B complex, the predominate transcription factor for IL-6 [75, 76]. Similarly, neuronal IL-10 inhibited LPS-activated microglial expression of IL-12, TNF-*α*, and Nitrous Oxide (NO), as well as complement synthesis [48]. Evidence for neurons having an inhibitory effect upon microglial phagocytosis was demonstrated by showing increased apoptosis of microglia after exposure to neuron conditioned media [77]. The increased expression of the semaphorin Sema3A, by neurons, induced apoptosis in activated microglia preventing them attacking neighbouring neurons [78]. Furthermore, the expression by neurons of both CD45 and CD22 was found to inhibit expression of inflammatory cytokines by microglia and provided further evidence for neuronal regulation of the innate immune response [78, 79]. A direct immunoregulatory effect of neurons upon microglia is also shown by the up regulation of MHC II expression after local neuronal activity was blocked [80, 81]. 

## 9. The “Death Signal Response” of the Innate Immune System Is Protective Because It Initiates Apoptosis and Promotes Tissue Homeostasis

Acute brain damage due to ischemic infarction results in both primary necrotic cell death and the formation of apoptotic cells [12, 82, 83]. If apoptotic cells are not rapidly cleared, they will accumulate and release neurotoxic proteins into the host tissue to produce the so-called secondary cell death and further tissue damage [11, 84, 85]. 

The induction of apoptosis in infiltrating T cells and damaged neurons is a protective component of the “double-edged sword”. This renders apoptotic cells safe and provides the brain with a degree of immunosurveillance, by down regulating inflammation and promoting their clearance [86, 87]. The rapid clearance of apoptotic cells from areas of damage is therefore essential to promote tissue homeostasis [5, 11, 88].

Active apoptosis of infiltrating T lymphocytes is induced by neurons and glia utilising the “death signalling pathways” based upon members of the TNF super family and include CD95(FasL)/CD95 (Fas) and the TNF-lymphotoxin receptor-TNF receptor-1 (TNRF-1) [89–93]. The role of TNF/TNFR death signalling pathway is more related to inflammatory signalling, whereas the CD95(Fas)/CD95Fas L pathway is considered to be more closely involved with induction of apoptosis [94, 95].

The initiator of apoptosis, CD95L, is expressed by neurons, astrocytes, and oligodendroglia and transmits an apoptotic signal to target T cells following ligation by either an agonistic antibody or ligands CD95L and TNF-related apoptosis inducing ligand (TRAIL) [89, 96, 97]. Under hypoxic conditions, the death signalling pathway Fas/CD95/apo-1 functions as a death receptor and is responsible for triggering apoptosis in ischaemic neurons, transforming them into “safe” targets for phagocytic clearance [92]. The interaction at the cell surface between CD95/CD95L induces the activation of caspases and subsequent apoptosis of the target cell. For example, apoptosis is initiated in activated T lymphocytes, resulting in their subsequent engulfment and clearance by microglia, leading to a down regulation of the inflammatory response [98].

## 10. Apoptotic Cell Clearance: An Anti-Inflammatory and Protective Role for the CNS Innate Immune System

The clearance of apoptotic cells expressing ACAMPs by phagocytes of the innate immune system (predominantly microglia) is vital to prevent their accumulation and subsequent release of neurotoxic molecules [11]. The phagocytosis of apoptotic cells by glia is regarded as “nonphlogistic” because it is associated with inhibition of proinflammatory cytokine expression and down regulation of inflammation [99–101]. Phagocytosis of apoptotic cells is associated with the release of TGF-*β*, IL-10 and tissue growth factors such as VEGF. All these molecules are capable of stimulating tissue repair and regulating CNS inflammation [100–103]. Recovery from EAE is increased through induction of apoptosis in inflammatory T cells by the TNFR signalling pathway [86, 87]. In TNFR knockout mice, T cell apoptosis is reduced by fifty percent in the periphery of demyelinating plaques [94].

Apoptotic cells are recognized as “altered self” because they express surface molecules termed apoptotic-associated molecular patterns (ACAMPS) [8, 28, 104, 105]. Mannose sugars, oxidized low-density lipoproteins, and electrical charge have all been proposed as ACAMPS; however, the best characterised to date is the phosphatidylserine lipid molecule (PS) [104]. Glia and macrophages express a range of phagocytic receptors (PR) that recognize ACAMPS including the PS-R, CD 14, CD36, milk fat globulin (MFG-EGF 8), and triggering receptor expressed by myeloid cells-2 (TREM-2) [30, 84, 104–111].

Activation of the classical C pathway through the first C component, C1q, recognizing ACAMPS, initiates the generation of opsonins C3 and C3b [112, 113]. These two opsonins enhance phagocytic clearance of apoptotic cells, because they are recognized by microglia expressing the CR3 and CR4 receptors [29, 33, 114, 115]. The detection and clearance of apoptotic cells by the innate immune system is therefore vital for the promotion of tissue homeostasis as it regulates the protective component of the CNS innate immune response [86, 87].

## 11. The Complement Pathway Has a Pivotal Regulatory Role in the CNS Innate Immune Systems “Double-edged Sword” Response

The complement system is an integral part of CNS innate immune system and comprises of three pathways, the classical (CP), alternative (AP), and lectin pathway. Each pathway is composed of soluble and surface proteins expressed by almost all cell types with both neurons and glia expressing the full range of complement pathway proteins [31] (see Figure 2). The classical pathway is activated by hypoxic neurons, myelin debris, DNA, various neurotoxic proteins, and apoptotic cells all binding to the first C component C1q [33, 116, 117]. C1q represents a PRR and is closely involved with the clearance of apoptotic cells and toxic debris from injured CNS. Microbes activate the alternative pathway by binding to C3 to promote C5 formation and subsequent membrane attack complex (MAC) formation. The lectin pathway is not regarded as an important factor in CNS inflammation **(**see Figure 2).

Activation of the CP and AP pathways generates C3 with subsequent production of C3b and iC3b, two opsonins that target apoptotic cells and promote their clearance by macrophages and microglia expressing the CR3 (CD11b/CD18) and CR4 (CD11c/CD18) receptors [8, 114, 115]. These two receptors are also located on activated microglia and the Kolmer cells of the choroid plexus [118]. These two cell types are responsible for clearing neurotoxic debris and apoptotic cells from the CSF, emphasizing the importance of the innate immune system for removal of debris and apoptotic cells from the ventricle in the acutely injured brain [29, 33, 115].

Both alternative and classical CP converge to produce the cytolytic terminal membrane complex C5-9(MAC) which produces cell lysis and tissue injury. Brain cells are particularly vulnerable to C attack and express a wide range of RCAs to inhibit local C3 and MAC synthesis in order to maintain tissue homeostasis [118–121].

Activation of the CP results in the formation of two anaphylotoxins C3a and C5a that are capable of acting as chemoattractants to glial and myeloid cells expressing the receptors, C3aR and C5aR [31, 32]. However, C3a has recently been shown to have an immune-regulatory function by inhibiting proinflammatory cytokines and by its capacity to reduce NMDA-induced neuronal death [122, 123]. Further evidence, discussed below, describes how the generation of C3a contributes to stem cell chemotaxis into areas of inflammation, potentially enhancing tissue repair [124].

## 12. The Regulators of Complement Activation Proteins (RCAs) Have Multiple Protective Roles Preventing Inappropriate Complement Attack

To prevent “self -destruction” and reduce tissue injury, the CP are regulated by proteins described as regulators of complement activation (RCAs). These regulators are divided broadly into membrane and fluid phase proteins (for detailed review see [119]). The membrane-related RCAs include CR1(CD35), DAF (CD55), and CD46 (MCP). These three regulators block the classical and alternative pathways at the C3/C5 convertase stage. CD59 blocks formation of the MAC at the common terminal pathway stage of both the CP and AP pathways [125–127]. The fluid-phase RCAs include C1inhb, the inhibitor that regulates C1 activation in the classical pathway Factor H (FH) prevents factor B from binding to C3b and this inhibits the C3/C5 convertase step in the classical pathway. Clusterin and protein S both prevent C5b-7 formation in the terminal pathway, reducing the extent of MAC driven inflammation [31, 32, 119]; see Figure 2. Not only do the RCA regulate C pathway activation, but they also have multiple protective roles as defined in the following 4 subsections.

### 12.1. RCA Regulate the CNS Innate Immune Response and Reduce Brain Inflammation

Transduction of complement and neurotoxic proteins through the disrupted BBB will contribute to the activation of the potentially cytolytic C components on the cell membranes of neurons and glia. Following head injury and ischemic stroke complement mediated neuronal damage has been reported and this corresponds to local C synthesis by neurons and glia [31, 32]. Deficiency of the RCAs CD55, CD59 and FH have all been shown exacerbate the severity of inflammation in Experimental Autoimmune Encephalomyelitis (EAE) [128, 129].

Neurons and neuronal cell lines activate the C pathway resulting in MAC-induced cytolysis because *in vitro* they express low levels of the RCAs (CD59, CD46, DAF, and CR1) and (CD55) [120, 121, 130]. Factor H was the main neuronal regulator for C, but was present at low levels, as were the other fluid phase regulators Sp, clusterin, and Ci inhibitor [128–132]. *In vivo*, however, van Beek found that CD55 was in fact an effective “neuroprotective RCA” in chronic, but not in acute CNS inflammation [121].

Astrocytes and microglia express a full range of RCAs (CD46, CD59, DAF, FH, and clusterin), effectively protecting themselves against bystander C attack in areas of tissue damage and inflammation [125, 127, 128, 133–136]. However, in human oligodendroglioma cell lines CD59, MCP and DAF(CD55) are all expressed, together with the regulators of the alternative C pathway C1inhb, FH, S protein, and clusterin [136, 137]. Overall, neurons express low levels of RCAs and are vulnerable to C attack, whereas astrocytes, microglia, and oligodendrocytes are better placed to support tissue repair, because they are protected by a range of RCAs against attack by C activation. This property increases glial survival in areas of tissue damage, together with glial providing important support for neuronal sprouting through the expression of clusterin [138]. These data emphasis the therapeutic potential of manipulating glial expression of RCA in order to minimise neuronal injury by regulating the hosts' inflammatory response.

### 12.2. RCAs Have Immunoregulatory Functions in the Adaptive Immune System Reducing Brain Inflammation

The range of immunoregulation provided by the RCA has recently been extended to include the down regulation of systemic B and T cell activity. This regulatory property of RCAs coordinates the regulation of both the innate and adaptive arms of the immune response reducing the inflammatory response in the CNS [139–141].

The membrane bound RCA, CD46, binds to C3b and this in turn stimulates Treg that inhibit the activity of other T cells [141, 142]. CD55 and CD59a also regulate T cells by reducing the stimulatory effects of C on both T cells, antigen presenting cells (APC) and B cells [139, 142]. The exact mechanism responsible for RCA regulation of T cell activity is not yet understood, but CD59a has a postulated direct inhibitory effect upon APC independently of complement. Conversely in EAE, DAF (CD55) suppression of T cell activity was dependent upon C pathway integrity which was responsible for reducing the expression of the inflammatory cytokines IFN-*γ* and IL-2 [140]. Therefore, the presence of RCAs in acutely injured and inflamed tissues not only reduces C activation, but also regulates the adaptive immune response by inhibiting T cell proliferation and reducing inflammatory cytokine expression. Despite this evidence for the inhibitory effects of the individual CRP regulators on T cell activity, the exact mechanism responsible for this effect is yet to be determined [142].

### 12.3. RCAs Are “don't eat me” Signals Indicating “Self” and They Are Lost during Apoptosis

One strategy for evading detection by microglia and preservation of tissue homeostasis is the expression of a group of molecules that define self by acting as “don't eat me” signals, the so-called “self-associated molecular patterns” (SAMPS) [8, 9]. A universal example of a “don't eat me” signal (SAMP) is MHC-I which is present on host cells helping to define “self” and preventing their detection by natural killer cells [143]. The expression of “don't eat me” signals by host cells is therefore crucial for maintaining tissue homeostasis. For example, the RCA, CD46, represents “don't eat me” signal on host cells, but it is down regulated on the surface of apoptotic cells (“altered self”). This loss of CD46 on apoptotic cells promotes opsonisation with C3 and iC3b and facilitates their phagocytic clearance [144]. Furthermore, the presence of the RCAs FH, CD46 and CD55, all act as “don't eat me” signals on host cells. The presence of these “don't eat me” molecules prevents inappropriate attack by microglia against host cells with the preservation of tissue homeostasis [8, 11, 128, 144].

### 12.4. RCAs Interact with Sialic Acids Representing “Self” and This Inhibits Microglial Phagocytosis

An important marker of normal or host cells is glycoproteins that terminate with sialic acids and represent markers of “self” [145]. These sialic rich molecules are recognized by FH as representing a “don't eat me” signal and this interaction prevents phagocytosis of host cells by microglia [8, 9, 128]. One group of receptors known as siglecs also bind to the sialylated glycoproteins and contain the immuno receptor tyrosine-based inhibitory motifs (ITIMS) that inhibit microglial function again preventing inappropriate destruction of host tissue [145, 146]. The absence of sialic acids on pathogens and apoptotic cells represents a missing “self-signal” and promotes the phagocytosis and clearance of pathogens and apoptotic cells with reduction of proinflammatory cytokine expression [28, 84, 98, 146].

## 13. Neurons Express Neuroimmunoregulatory Molecules (NIRegs) to Preserve Tissue Homeostasis and Promote Survival during Inflammation

**Figure 3:**
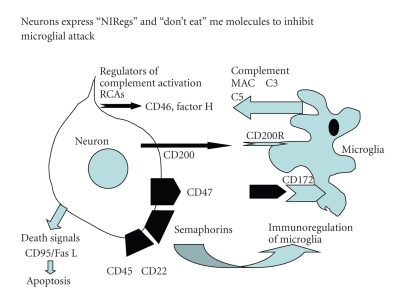
Neurons express a wide range of “selfdefence” proteins and receptors that reduce inappropriate bystander attack from activated microglia. Semaphorin 3a and CD22, CD45 all reduce proinflammatory cytokine expression and inhibit microglial activity. The neuro immunoregulatory (NI Regs) CD200 and CD47 that interact with the their counter receptors CD200R and CD172 on microglia and myeloid cells, reducing microglial activation (Black arrows). Regulators of complement activation RCAs) such as CD46 also act as “don't eat me” signals preventing attack from host microglia. Neurons also express “Death signals” to initiate apoptosis in damaged cells or infiltrating T cells making them targets for phagocytosis.

To help neurons and other host cells evade detection by activated microglia and macrophages, they express a group of “don't eat me” signals termed neuroimmunoregulatory molecules (NIRegs) [6, 8, 10, 24, 25]. These molecules reduce the severity of any inflammatory response by inhibiting microglial phagocytosis. The range of NIRegs regulating microglia activity is expanding and includes CD200 (and its receptor CD200R), the integrin CD47 with its receptor CD172, together with the semaphorin Sema 3A and CD22 [37, 39, 40, 78, 79]. The down regulation of the expression of NIRegs, CD200 and CD47, promotes microglial activity as found in demyelinating plaques from cases of multiple sclerosis [37]. See Figure 3.

## 14. CD200-CD200R: An NIReg Pathway

CD200 is a 41–47 kd surface molecule and a member of the Ig supergene (IgSF) family characterised by two IgSF domains that represent the most commonly found domain type in the leucocyte membrane [40]. The presence of two IgSF domains suggests that this molecule is related to cell adhesion and regulation. As a glycoprotein, CD200 is located on the membrane of myeloid cells, cerebellar neurons, retinal neurons, and vascular endothelium [35, 37–40, 147]. The counter receptor to CD200, CD200R, also contains two IgSF domains and is expressed by myeloid cells and rodent brain microglia [36, 147, 148]. 

In CD200-deficient mice, the number of activated microglia and macrophages was more numerous after an experimental lesion, as compared with the wild type animal. This evidence demonstrated that the CD200-CD200R interaction regulated microglial activation and inflammatory cell trafficking across the BBB [36, 147]. This observation is consistent with the finding that CD200^−/−^ mice have spontaneously activated microglia and are highly susceptible to induction of experimental allergic uveitis [147]. Expression of CD200, but not CD200R, was reduced in and around demyelinating plaques in multiple sclerosis (MS) allowing unrestrained microglial activation, although individual astrocytes expressing CD200 have recently been demonstrated in MS and are regarded as neuroprotective [37, 148]. Overall, the CD200 level was reduced in MS tissue as compared with normal tissue, indicating a failure of the CD200-CD200R pathway in this inflammatory CNS disease [37, 148].

The expression of CD200 is an important immunoregulatory signal during apoptosis because it is under the control of both P53 and caspase-dependent pathways. CD200 expression is increased on the surface of apoptotic cells and because of its immunosuppressive properties this inhibited proinflammatory cytokine expression by apoptotic dendritic cells *in vitro*. The presence of CD200 on apoptotic cells also reduced the severity of tissue damage because of its inhibitory interaction with microglia expressing its counter receptor CD200R [149].

## 15. CD47-CD172 a Further NIReg Pathway Present in the CNS

As a member of the IgSF protein family, CD47 is constitutively expressed by endothelium, neurons, macrophages, and dendritic cells [39, 46, 150, 151]. CD47 has five trans-membrane regions with alternatively spliced isoforms of CD47 having a tissue specific expression; isoform 2 is present in bone marrow, whereas isoform 4 is highly expressed in brain [151]. The counter receptor for CD47 is signal regulatory protein SIRP alpha (CD172), a plasma membrane protein with three Ig domains in its extra cellular component; it is expressed by myeloid cells and neurons [39]. The interaction between CD47 on a host cell with a myeloid cell expressing CD172a recruits the tyrosine phosphatases SHP-1 and SHIP-2 resulting in the down regulation of macrophage phagocytosis, the prevention of neutrophils migrating across the BBB, an increase of TGF-*β*, expression and a reduction of interferon *α* levels all contributing to the reduction of the severity of any inflammatory response [46, 151, 152]. CD47 also interacts with a further counter receptor, thrombospondin TSP, expressed by microglia, astrocytes, and smooth muscle cells [153]. The TSP molecule acts as a bridge between the apoptotic cells and the phagocyte. TSP binds to CD47 expressed on neurons and T cells and this interaction promotes apoptosis through the death signal CD95/Fas pathway. In the case of TSP binding with CD47 located on activated T cells, the severity of tissue inflammation is reduced by this interaction, because it promotes T cell apoptosis and clearance [153].

Cells deficient in CD47 are rapidly cleared from the systemic circulation by the spleen indicating the presence of CD47 represents a “don't eat me” signal and prevents phagocytosis of host cells [154]. For example, apoptotic cells loose surface CD47 which reduces their ability to phosphorylate CD172a, removing their inhibitory effect upon local microglia and enhancing their own clearance from areas of tissue damage by phagocytosis [155]. The immunoregulatory role of CD47 is emphasised in human disease because this NIReg is lost at the edge of a demyelinating plaque in multiple sclerosis, removing the immunoregulation of local microglia and increasing disease progression [37, 148].

## 16. The Interaction between Brain Stem Cells and the Innate Immune Response to Brain Injury

Neural stem/precursor cells (NPCs) have not only a well-defined role providing replacement for damaged neurons, but also a range of beneficial properties termed “therapeutic plasticity” which include the expression of neuroprotectants and immunoregulatory molecules [156]. Stem cells differentiate into a glial-like cell with inherent regulatory and protective activities capable of rescuing dying neurons and oligodendrocytes [4, 157, 158]. The range of protective properties (therapeutic plasticity) includes the expression of neuroprotective and immunoregulatory molecules, a concept termed the “bystander or chaperone” effect [156, 159]. Amongst the protective effects expressed by stem cells is the potential to regulate the local innate immune and adaptive systems and as a consequence promote tissue homeostasis [5, 156, 160].

## 17. Stems Cells Down Regulate Local Inflammation to Promote Their Restorative Properties

Neural stem cells (NSC) are located in the subventricular zone (SVZ) and renew to produce neurons and glia [161, 162]. NSC introduced into the systemic circulation are remarkably resilient to destruction by inflammatory cells of the adaptive immune system. However, NSC are susceptible to T cell-mediated killing because they express both costimulatory molecules CD80 and CD86 [163]. Stem cells are also able to divide into glial-like cells with “regulatory” and “protective” activities that support dying neurons and oligodendroglia, a function mediated by the expression of growth factors and immunoregulatory molecules that control the local innate immune response, a characteristic described as “therapeutic plasticity” [15].

## 18. Stem Cells Are Able to Immunoregulate T Cells

**Figure 4:**
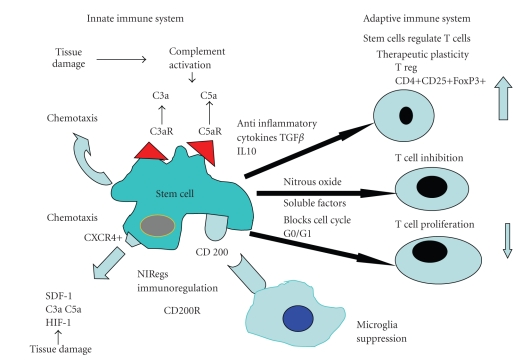
The figure shows the interactions between stem cells expressing the CXCR4 receptor and stroma-derived factor SDF-1 with the complement anaphylotoxin chemoattractants C3a and C5a; tissue damage contains the chemoattractant Hypoxia factor (HF-1), thrombin and complement anaphylotoxins (C3a and C5a). Stem cells express the (NIReg) immunoregulatory signal CD200 that inhibits microglial activation via the counter receptor CD200R. T cells are also inhibited through several different inhibitory pathways expressed by stem cells as well as stimulating Tregs; these interactions all reduce the severity of the inflammation and stimulate stem cell migration into areas of inflammation to assist with tissue repair; these effects are termed “therapeutic plasticity”.

The concept of a “regulatory” glial stem cell controlling the local CNS innate immune response shares similarities with the same role carried out by the T regulatory cells (CD4+CD25+FoxP3+) in the adaptive immune system [8, 15]. This small population of T cells known as T regs suppress the T lymphocyte response by expressing TGF-*β* and IL-10, both of which can expand the Treg population and also inhibit the proinflammatory cytokine expression by microglia [73–75, 164–166]. The mechanism responsible for T cell-based neuroprotection is not entirely clear, although lymphocytes express a range of neurotrophic growth factors, including brain-derived neurotrophic factor and ciliary trophic growth factor, as do astrocytes and macrophages [73–75, 167–171]. See Figure 4.

Emerging data indicate that mesenchymal stem cells (MSC) are capable of immunoregulating inflammation through T cell suppression. In acute experimental cerebral ischaemia and EAE, MSC inhibited activated T cells and stimulated the expansion of T regs as demonstrated in chronic inflammatory diseases such as rheumatoid arthritis and colitis [7, 17, 172, 173]. The mechanism responsible for this immunosuppressive effect of MSC upon T cell proliferation is not clear, but the cytokines IFN-*γ*, TNF-*α* ILI-*α*, and IL-*β*2 were all implicated in a complicated regulatory pathway between T cells and MSC. *In vitro* studies have found that MSC do not suppress activated T cells unless the T cells are themselves producing the key proinflammatory cytokine IFN-*γ*. Low levels of this cytokine in combination with TNF-*α* IL-I*α* and IL-*β*2 promote MSC-related T cell suppression as confirmed by MSC from mice deficient in IFN-1 receptor being unable to inhibit T cell proliferation [174, 175]. This data indicate that an initial low level of T cell IFN-*γ* expression is required before the MSC can inhibit T cell proliferation. The proinflammatory cytokines IFN-*γ*, TNF-*α* IL-I*α*, and IL-*β*2 are responsible for T cell inhibition, because they promote iNOS (inducible primarily in macrophage nitric acid oxidase) and eventually NO expression by MSC. Both NO and the proinflammatory cytokines expressed by MSC are postulated as molecules that mediate the suppression of T cells. The central effect of NO in this regulatory pathway is confirmed in mice lacking iNOS, because MSC from these animals are not able to immunosupress T cells [174, 175]. 

## 19. Stem Cells Are Able to Migrate into Areas of Tissue Injury and Inflammation Where They Regulate T Cell Activity

A further immunoregulatory property of MSC is dependent upon their ability to migrate into areas of tissue damage and express the leukocyte chemokines CXCL9, CXCL10, and CXCL11. All of which are ligands for the T cell-specific chemokine receptor CXCR3 [175]. The close proximity of MSC to T cells is vital for NO to have its immunosuppressive effect, because NO is only effective over short intercellular distances. If the CXCR3 receptor is inhibited, the immunosuppressive effect of MSC is lost, because these stem cells will not be able to migrate into tissues containing T cells [175]. In addition, an important immunoregulatory characteristic of MSC is the initiation of T cell apoptosis; this effect is absent when MSC from either iNOS^−/−^ or IFN*γ* ^−/−^ mice are cocultured with activated T cells [174, 175].

Further evidence for stem cell regulation of T cell proliferation has been shown by mesenchymal stem cell inhibition of the T cell cycle at the G0/G1 phase, preventing the clonal expansion of activated T cells. [176] More recently, the NIReg CD 200 which has been located on both normal human cancer stem cells (including malignant brain tumors such as the glioblastoma) provides a signalling pathway to allow stem cells present within inflammation and tumours to evade immunodetection and consequently thrive [177]. See Figure 4. 

## 20. Stromal Cells, Niche Formation and the Regulation of the CNS Innate Immune System

In the systemic organs, stem cell renewal and progenitor differentiation are regulated by stromal cells located in specialized microenvironment termed a “niche” [4, 161, 178]. Stromal cells express a range of markers including vimentin, laminin, fibronection, osteopontin, and variably STRO-1, VCAM-1, endoglin, and MUC-18/CD146. Soluble factors such as Stroma-derived factor 1 (SDF-1) that signal between stromal and stem cells and are capable of regulating stem cell renewal and differentiation within the niche [178].

In the adult mouse, brain stem cells, with a characteristic of astrocytes, are located in two discrete niche areas, the subventricular zone (SVZ) of the lateral ventricle and subgranular area of the hippocampus (SGZ) [4, 161, 162, 179]. Outside of these two areas, astrocytes do not appear to have neurogenic properties. The stromal cells in the mouse stem cell niche SVZ have endothelial and potentially ependymal charcteristics as indicated by their expression of SHH, Notch, Wnt, TGF-*α*, FGF and VECF molecules [4, 178, 180].

In the brain, fibroblasts, surrounding blood vessel walls and ependymal cells are both regarded as stromal cells, because they provide a niche to control adult neurogenesis and are immunoregulatory cells [162, 181]. Ependymal cells are present in the SVZ, but following injury they switch to the radial or chaperone phenotype and migrate into sites of injury and inflammation in order to prepare the ground for the migration of “protective” NSC [4, 6]. Ependymal cells also express a range of molecules Notch I, bone morphogenic proteins, GPCR for the C anaphylotoxin C3a that increased their response to the protein SDF-1. This protein is expressed by ependymal cells and responsible for regulating neuro- and gliogenesis [162, 182, 183]. SDF-1 is also increased in areas of tissue damage and functions as a chemoattractant to a variety of stem cells that express the G protein-coupled, transmembrane, cytokine receptor CXCR4 [87]. CXCR4 is positively regulated by the tissue hypoxia inducible factor (HIF-1), TGF-*β*, IL-4, and IL-7: all of these molecules are present in damaged or inflamed tissues [183]. SDF-1 is also increased in myocardial and brain ischaemic infarction, underlying the possibility that the SDF-1—CXCR4 pathway is important for attracting stem cells into areas of damage to promote tissue repair [184, 185]. Interestingly, SDF-1 is down regulated by two well-defined anti-inflammatory molecules TGF-*β* and steroids, whereas thrombin, fibrinogen, and C3a are all found in areas of inflammation and increase the chemotaxis of CXCR4+ stem cells to low dose SDF-1 [183, 186]; see Figure 4. 

Recent evidence has also underlined the importance of the C pathway for the trafficking of haemopoietic stem cells from bone marrow into blood and damaged tissue [183]. Activation of C results in the formation of C3a and this functions as a target to sensitise CXCR4+ stem cells to high levels of SDF-1, as found in areas of inflammation and promotes stem cell entry into these areas of tissue damage [183, 187]. This stem cell chemoattractant response to C3a and C5a was blocked by C3aR and C5aR inhibitors, respectively [124]. A further relationship between the C pathway and regeneration is the protective effect of CD55, an RCA, promoting neuronal sprouting [121, 188].

## 21. Stem Cells Regulate the Severity of CNS Inflammation by Systemic Immunosuppression

Stem cells have been shown to contribute to both immunoregulation and neuronal protection in both chronic and acute CNS infection and ischemia [17, 172, 189–192]. The immunoregulatory effect of NCS was observed in one study involving the initiation of EAE. The administration of intravenous NSC inhibited the peripheral T cells within lymph nodes and as a consequence reduced the severity of EAE [15]. A similar experiment with mesenchymal stem cells MSC also reduced the severity of chronic EAE through peripheral immuno suppression [192].

More recently in an experimental model of acute cerebral stroke, intravenous injection of neural/stem cell precursor (NPC) produced a profound antiapoptotic and anti-inflammatory effects. This included the down regulation of TNF-*α* and IL-6 in both CNS and lymph tissue resulting in a reduced volume of brain haemorrhage. [156]. These data show that peripheral stem cells reduce the severity of CNS inflammation by regulating entry of systemic anti-inflammatory cytokines through the open BBB following haemorrhage [156]. Alternatively, NPC enter the CNS as in the EAE model to express their own anti-inflammatory cytokines to stimulate immuno-regulation and increase their own survival [15, 156, 190, 191]. 

Rather than differentiating into the terminal stage to replace damaged neurons, NPC promote tissue repair by acting as bystanders expressing their “therapeutic plasticity” phenotype by producing anti-inflammatory cytokines and immuno regulators of T cells. These data indicate that peripherally administered stem cells can also regulate the CNS innate immune response through effects upon the systemic lymphoid system [156, 193].

## 22. Inflammation Can Have Protective Effects by Stimulating Bone Marrow Cell Survival in the CNS

Recent evidence has found that bone marrow cells gain access to the brain following disruption of the BBB and are capable of differentiating into microglia but not astrocytes [194]. The survival of bone marrow cells (BMS) when transplanted into brains with an acute meningitis due to *S. pneumoniae* infection was greatly enhanced and they rapidly differentiated into functional microglia contributing to the clearance of debris and apoptotic cells [195, 196]. Similarly, transplanted oligodendroglial precursors exposed to tissue inflammation were effective at remyelination [197, 198]. The presence of proinflammatory cytokines and activated microglia in host tissue with ischemic infarction, infection, and metabolic diseases has been shown capable of promoting BMS survival together with increasing microglial differentiation from the transplanted monocytes [6]. Therefore, successful colonization of CNS tissue by BMS cannot be assumed to always require down regulation of the innate immune system. The absence of tissue damage can prevent activation of the innate immune system which can under some circumstances act as a positive signal for tissue repair [198]. This interaction between host CNS inflammation and the enhanced survival of BMS provides an interesting therapeutic opportunity [6].

## 23. Conclusion

The balance between the protective and destructive effects of the innate immune response against pathogens and brain injury has been termed “a double-edged sword”. (Wryss coray 2002). This balance must be critically regulated in order to promote conditions supportive of brain repair and allow the damaged brain to return to normal function (homeostasis). 

The disruption of the BBB exposes neurons to potentially neurotoxic proteins from the systemic circulation. These proteins are recognized as “nonself” because they contain PPAMPS. This stimulates the CNS innate immune system to express “selfdefence” proteins including the defence proteins called serpins in order to counter the neurotoxic effects of the systemic proteins upon the brain. In acute brain injury, the presence of these “selfdefence” proteins acting rapidly to promote repair is of potential therapeutic importance.

The CNS innate immune system is capable of expressing “death signals” (CD95L/FAS/CD95FAS-L) to initiate apoptosis in damaged neurons and infiltrating T cells and rendering them safe targets for removal by the innate immune system. Therapeutic stimulation of these pathways represents a route to remove infiltrating T cells with reduction in the severity of CNS inflammation. 

The clearance of apoptotic cells is enhanced by the C opsonins C3band iC3b providing targets for clearance by phagocytic glial cells expressing various PRR that recognize these opsonins. The exact PRR responsible for the removal of apoptotic T cells, damaged neurons, and neurotoxic proteins is not yet known. The stimulus responsible for the selective expression of specific PRR by the individual cellular populations in the CNS innate immune system is therefore, an important future topic for research. 

Glia expression of C is closely self-regulated by RCA preventing bystander cell damage due to MAC attack of host and “nonself” targets. RCAs are not only regulators of C expression, but also suppress T cell activity reducing brain inflammation. However, the exact pathway responsible for this immunoregulatory effect remains unclear, but underlines the range of multiple immunoregulatory roles provided by this group of molecules. 

Host neurons and glia also express “don't eat me” signals and their presence prevents microglial attack; this is exemplified by a group of “don't eat me” signals called the NIRegs including the semaphorins, CD22, CD200, and CD47. The selective expression of these NIRegs provides several potential pathways for host cells and stem cells to evade the destructive effects of the innate immune response by reducing microglial attack of neurons and stem cells.

Stem cell replacement of damaged neurons represents a definitive response to acute brain injury, but recent evidence has shown that stem cells also exhibit “therapeutic plasticity”. This protective response includes the capacity to immuno-regulate tissue inflammation through anti-inflammatory cytokine expression, T cell inhibition, and expression of the NIReg CD200 that inhibits potentially destructive microglial activity. 

Brain stem cells expressing the CXCR4 cytokine receptor migrate into areas of inflammation and ischemia in response to the chemoattractant thrombin and the anaphylotoxins C3a and C5a. These two anaphylotoxins are expressed by glia and this underlines the potentially important relationship between stem cell survival and the protective component of the CNS innate immune system. It is likely that further molecules, expressed by the innate immune system, will be shown to have trophic properties towards stem cells, enhancing their survival in areas of tissue damage. Interestingly, the administration of peripheral stem cells into the systemic circulation has been shown to have immunoregulatory properties by reducing CNS injury and inflammation. These observations imply that the interaction between stem cells, T cells, and APC within local lymph node reduces the severity of CNS inflammation providing an accessible site, in the periphery, for the therapeutic manipulation of this neuroprotective effect.

Although many of these CNS immunoregulatory pathways are shared with systemic organs, they, nevertheless, represent potential therapeutic targets capable of regulating CNS inflammation and promoting stem cell survival. The elucidation of the immunoregulatory pathways shared between the CNS innate immune system and brain stem cells represents an important challenge, but one that is of great therapeutic potential, relevant to both acute brain repair and the restoration of tissue homeostasis.
